# Supplementary material for: Surface microlayer-mediated virome dissemination in the Central Arctic
Source: Microbiome. 2024 Oct 24;12:218. doi: 10.1186/s40168-024-01902-0 (PMC11515562; doi:10.1186/s40168-024-01902-0)
Supplement: Supplementary file 2 — Additional file 1. A .docx file with seven additional figures and more text information. [file 40168_2024_1902_MOESM1_ESM.pdf]

Supplement Material

Surface microlayer-mediated virome dissemination in the Central Arctic

Janina Rahlff[1,2,3*], George Westmeijer[1], Julia Weissenbach[1], Alfred Antson[4], Karin Holmfeldt[1]

[1]Centre for Ecology and Evolution in Microbial Model Systems (EEMiS), Department of Biology and Environmental Science, Linnaeus University, Kalmar, Sweden

[2]Aero-Aquatic Virus Research Group, Faculty of Mathematics and Computer Science, Friedrich Schiller University Jena, Jena, Germany

[3]Leibniz Institute on Aging - Fritz Lipmann Institute (FLI), Jena, Germany

[4]York Structural Biology Laboratory, Department of Chemistry, University of York, York, United Kingdom

***Corresponding author:** Janina.rahlff@uol.de

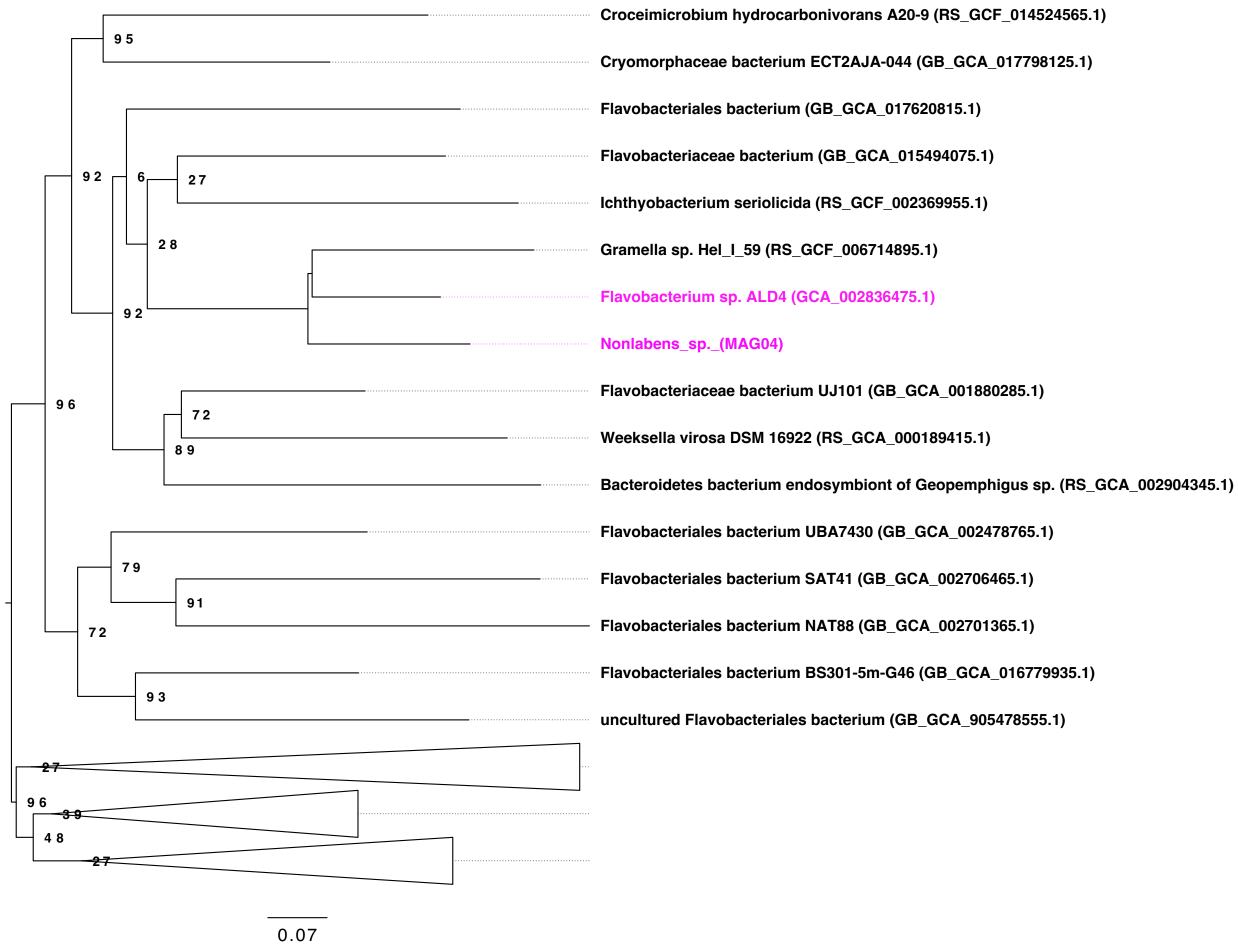

Figure S1: Tree showing phylogenetic relatedness genome "ext_mOTU_v3_31506" belonging to *Flavobacterium* sp. ALD4, to which melt pond sample reads mapped within the mOTUs tool and is related to *Nonlabens* sp. (MAG04) binned from the melt pond. The tree is derived from bac120.classify.tree (identification uses 120 bacterial marker genes) predicted by the classify_wf in GTDB-Tk v.2.1.0 [1] which uses pplacer v.1.1 [2] for the maximum-likelihood placement of genomes in the tree. A subnetwork of the full tree was extracted in Dendroscope v.3.8.8. [3] and the tree was rooted at the midpoint with the tips aligned in FigTree v.1.4.4 [4]. According to JSpeciesWS web server [5], ANIb for the two genomes is 67.31% with an aligned fraction of 28.05%.

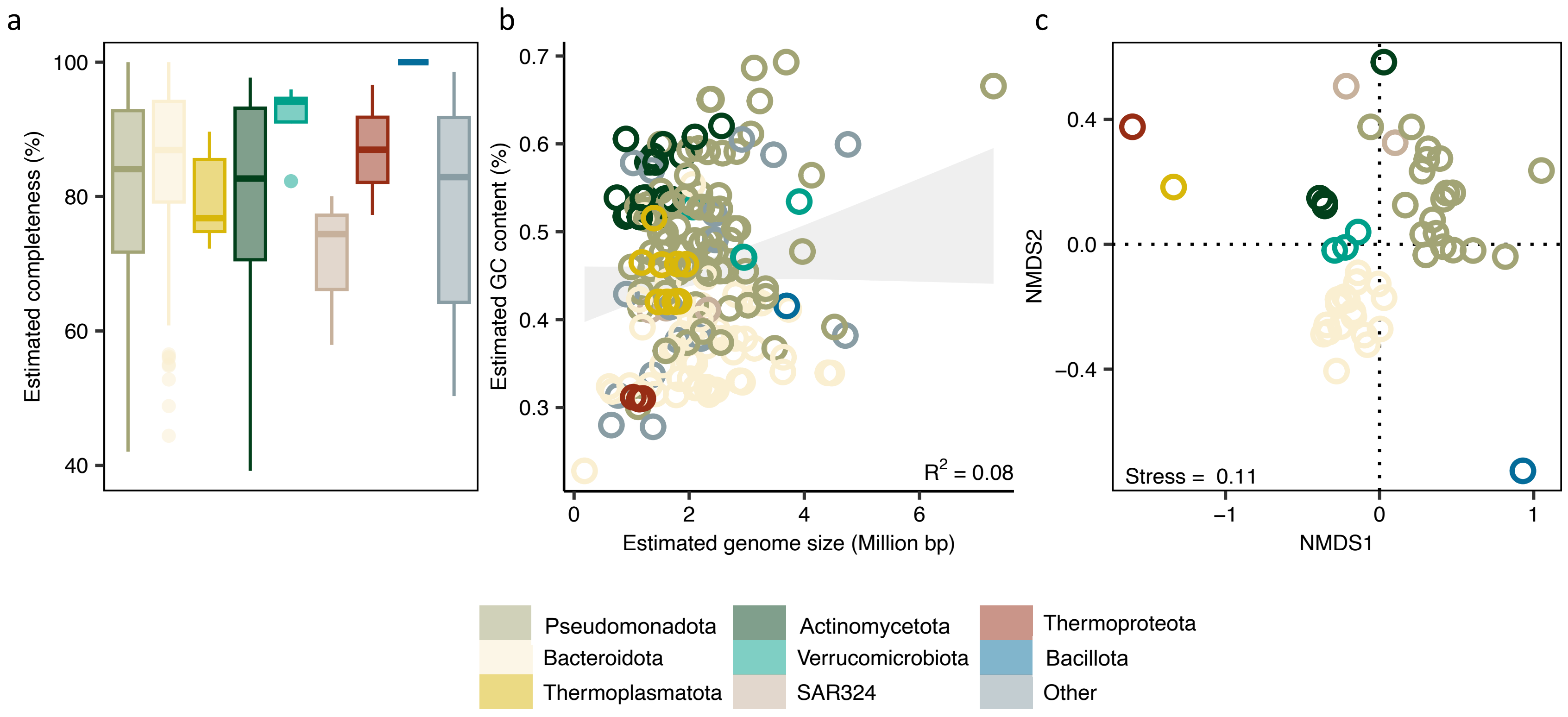

Figure S2: Characteristics of the MAGs. Estimated completeness grouped by phylum, based on CheckM2 [6] (a). Linear model of the estimated GC content (y) with the estimated genome size (x) revealing a low correlation ($R^2 = 0.02$, p-value = 0.34) (b). Divergence among MAGs based on functional orthologous genes (KO functional orthologs, accounting for multiple gene copies) using non-metric dimensional scaling (NMDS) (c).

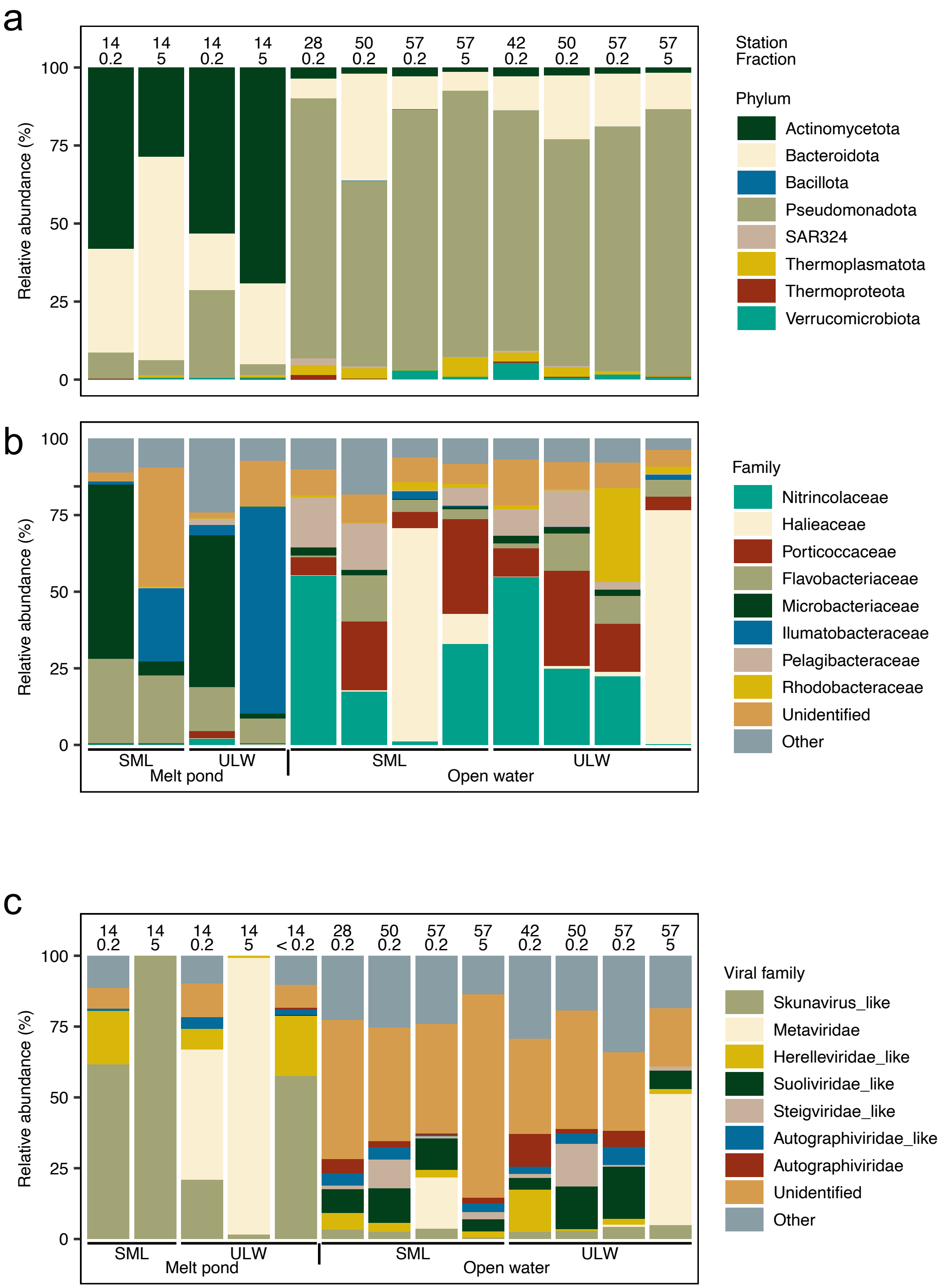

Figure S3: Taxonomic profiling expressed as relative abundance of the prokaryotic community based on metagenome assembled genomes (MAGs) at phylum (a) and family level (b). Relative abundances of viral families (c).

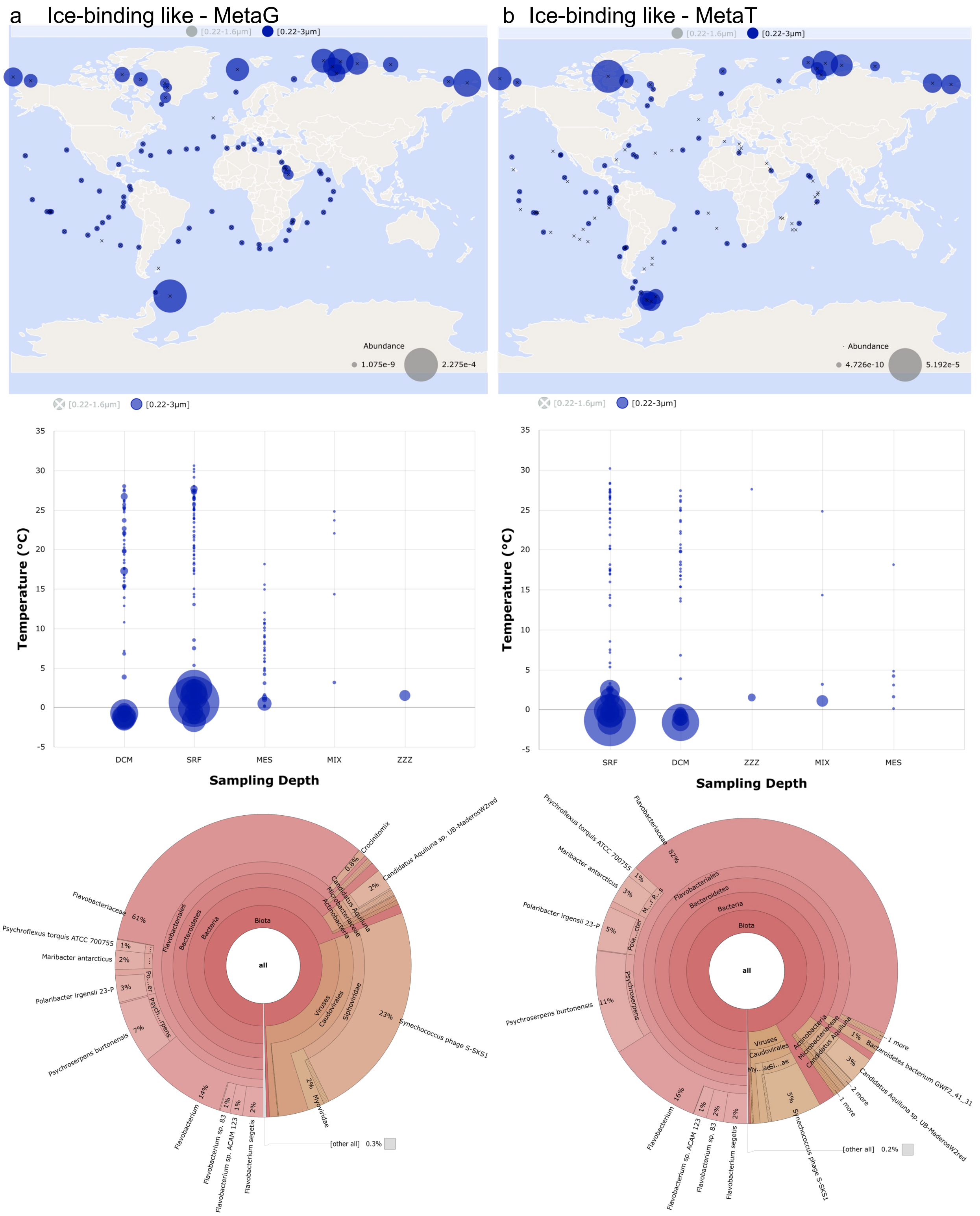

Figure S4: Biogeographic distribution, temperature range, and phylogenetic affiliation for the ice-binding like protein sequence based on Tara Ocean metagenomic (metaG) and transcriptomic

(metaT) datasets. Bubbles indicate abundances based on percent of mapped reads. Amino acid sequence in blastp corresponds to ORF134 of viral scaffold P24860_118_S16_metaspades_38_length_160796_cov_73||full. SRF = Surface water, DCM = Deep chlorophyll maximum, ZZZ = marine water layer, MIX = mixed layer, MES = mesopelagic zone

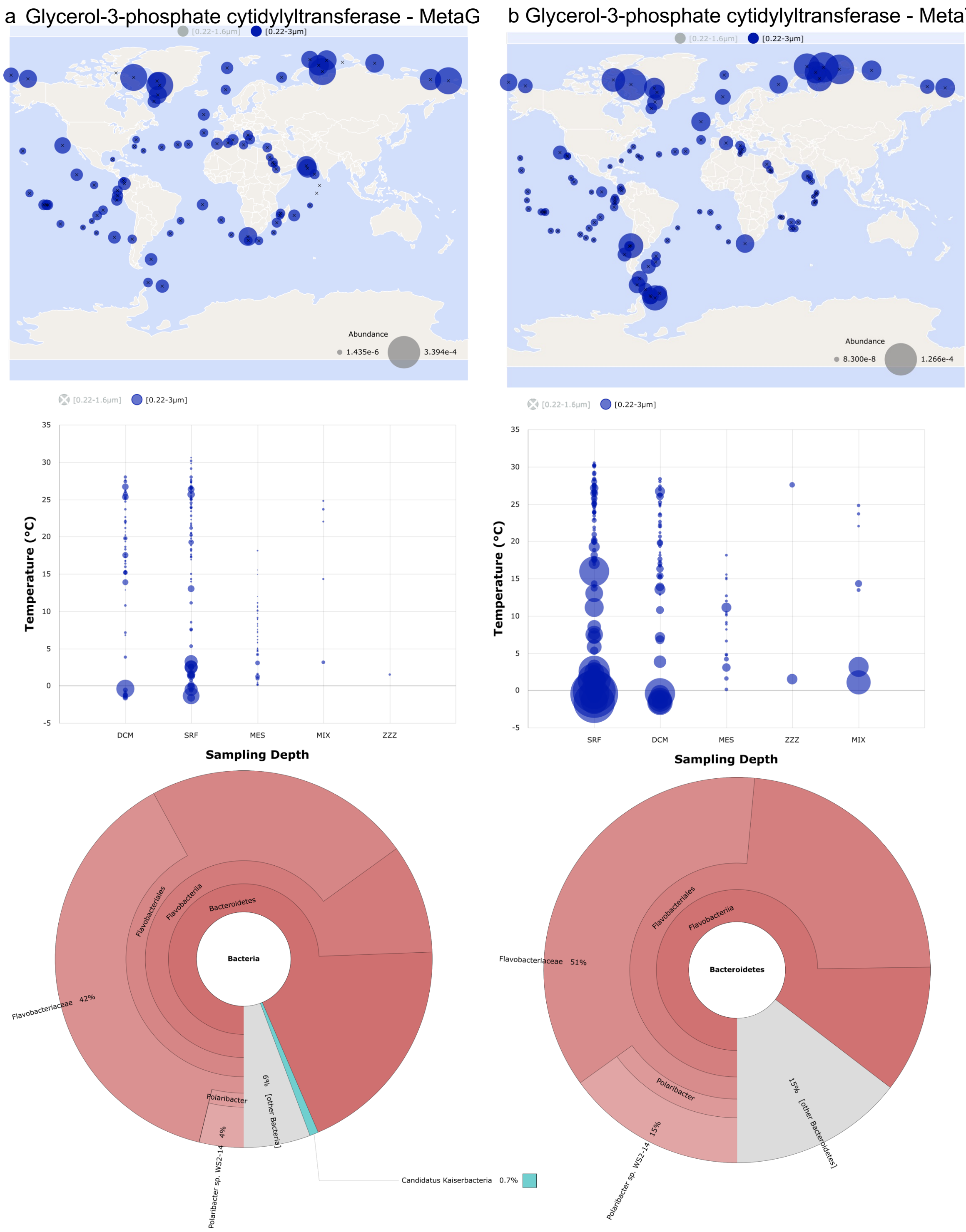

Figure S5: Biogeographic distribution, temperature range, and phylogenetic affiliation associated with glycerol-3-phosphate cytidylyltransferase sequence based on Tara Ocean metagenomic (metaG) and transcriptomic (metaT) datasets. Bubbles indicate abundances based on percent of mapped reads. Amino acid sequence in blastp corresponds to ORF39 of viral scaffold

P24860_114_S12_metaspades_256_length_95641_cov_7||full. SRF = Surface water, DCM = Deep chlorophyll maximum, ZZZ = marine water layer, MIX = mixed layer, MES = mesopelagic zone

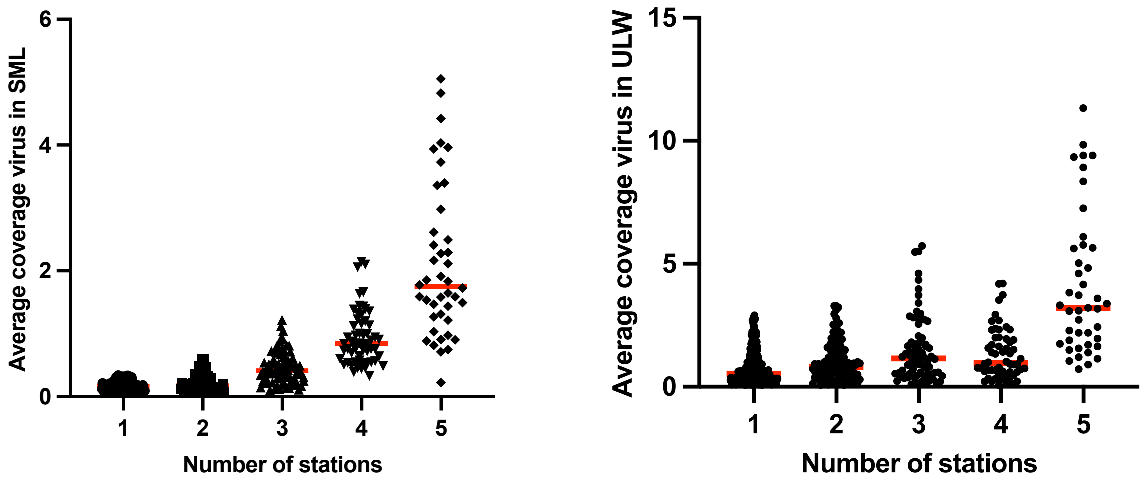

Figure S6: Correlation of the vOTU average coverage (red line indicates the median) for SML (a) and ULW (b) with number of stations a vOTU was present based on read breadth. This analysis shows that vOTU spread to different stations is more positively correlated to a higher vOTU coverage in the SML compared to ULW. The group comparison results are shown in the two tables below.

Dunn’s multiple comparison test results after Kruskal-Wallis test corresponding to SML groups from Figure S6 left plot

Dunn's multiple comparisons test	Mean rank diff.	Significant?	Summary	Adjusted P Value
1 vs. 2	-27.08	No	ns	0.7891
1 vs. 3	-114.9	Yes	****	<0.0001
1 vs. 4	-196.5	Yes	****	<0.0001
1 vs. 5	-240.9	Yes	****	<0.0001
2 vs. 3	-87.85	Yes	****	<0.0001
2 vs. 4	-169.5	Yes	****	<0.0001
2 vs. 5	-213.8	Yes	****	<0.0001
3 vs. 4	-81.61	Yes	***	0.0002
3 vs. 5	-126.0	Yes	****	<0.0001
4 vs. 5	-44.35	No	ns	0.4346

Test details	Mean rank 1	Mean rank 2	Mean rank diff.	n1	n2	Z
1 vs. 2	100.7	127.8	-27.08	107	92	1.757
1 vs. 3	100.7	215.6	-114.9	107	76	7.068
1 vs. 4	100.7	297.2	-196.5	107	58	11.12
1 vs. 5	100.7	341.6	-240.9	107	42	12.20
2 vs. 3	127.8	215.6	-87.85	92	76	5.229
2 vs. 4	127.8	297.2	-169.5	92	58	9.324
2 vs. 5	127.8	341.6	-213.8	92	42	10.59
3 vs. 4	215.6	297.2	-81.61	76	58	4.318
3 vs. 5	215.6	341.6	-126.0	76	42	6.043
4 vs. 5	297.2	341.6	-44.35	58	42	2.019

Dunn’s multiple comparison test results after Kruskal-Wallis test corresponding to ULW groups from Figure S6 right plot.

Dunn's multiple comparisons test	Mean rank diff.	Significant?	Summary	Adjusted P Value
1 vs. 2	-67.36	Yes	**	0.0076
1 vs. 3	-119.4	Yes	****	<0.0001
1 vs. 4	-111.6	Yes	***	0.0003
1 vs. 5	-303.9	Yes	****	<0.0001
2 vs. 3	-52.05	No	ns	0.6163
2 vs. 4	-44.23	No	ns	>0.9999
2 vs. 5	-236.5	Yes	****	<0.0001
3 vs. 4	7.819	No	ns	>0.9999
3 vs. 5	-184.5	Yes	****	<0.0001
4 vs. 5	-192.3	Yes	****	<0.0001

Test details	Mean rank 1	Mean rank 2	Mean rank diff.	n1	n2	Z
1 vs. 2	268.7	336.0	-67.36	357	116	3.367
1 vs. 3	268.7	388.1	-119.4	357	74	4.994
1 vs. 4	268.7	380.2	-111.6	357	58	4.210
1 vs. 5	268.7	572.6	-303.9	357	43	10.06
2 vs. 3	336.0	388.1	-52.05	116	74	1.869
2 vs. 4	336.0	380.2	-44.23	116	58	1.469
2 vs. 5	336.0	572.6	-236.5	116	43	7.077
3 vs. 4	388.1	380.2	7.819	74	58	0.2382
3 vs. 5	388.1	572.6	-184.5	74	43	5.140
4 vs. 5	380.2	572.6	-192.3	58	43	5.105

a)

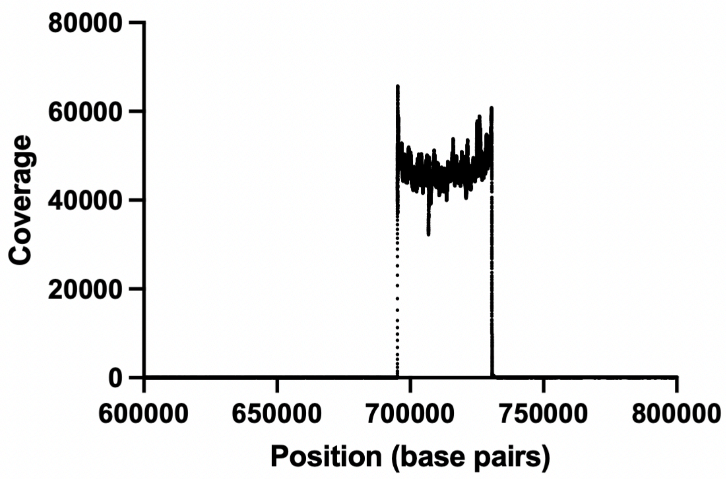

b)

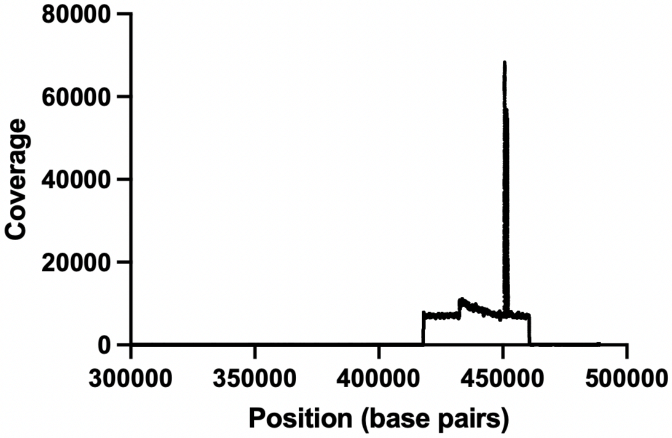

Fig. S7: Mappings of reads from sequenced DNA of the phage supernatant to the prophage carrying scaffolds of *L. aequorea* Arc30. Prophage 2, corresponding to Arctica_1 is located between position 694108 and 730554 bp (36.4 kb, a), and prophage 1, which corresponds to Arctus_1, between position 419014 and 463109 bp (44.1 kb, b).

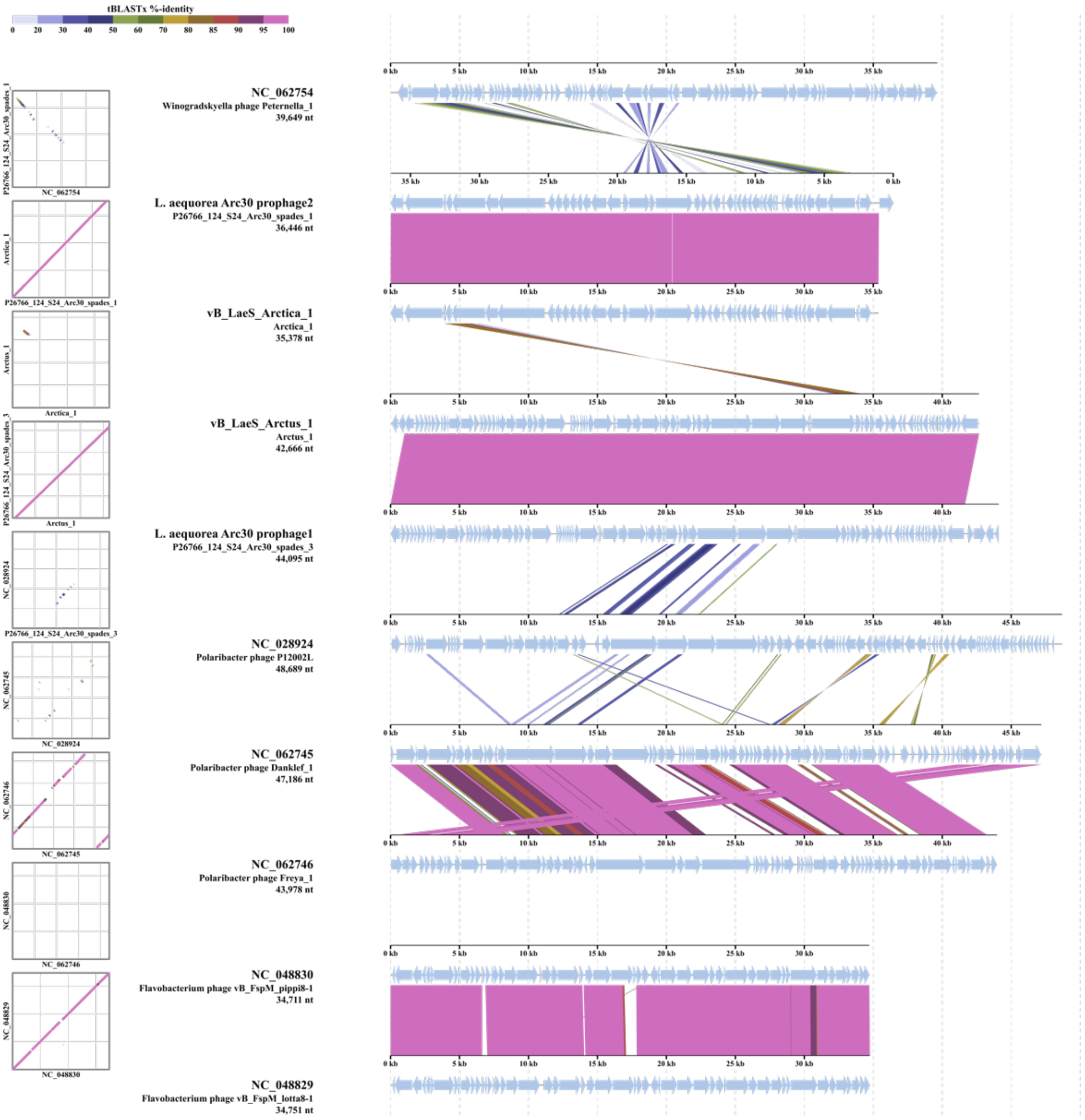

Figure S8: Alignments for vB_LaeS_Arctus_1 and vB_LaeS_Arctica_1 with related phages based on tBLASTx analysis conducted in VipTree v.4 [7].

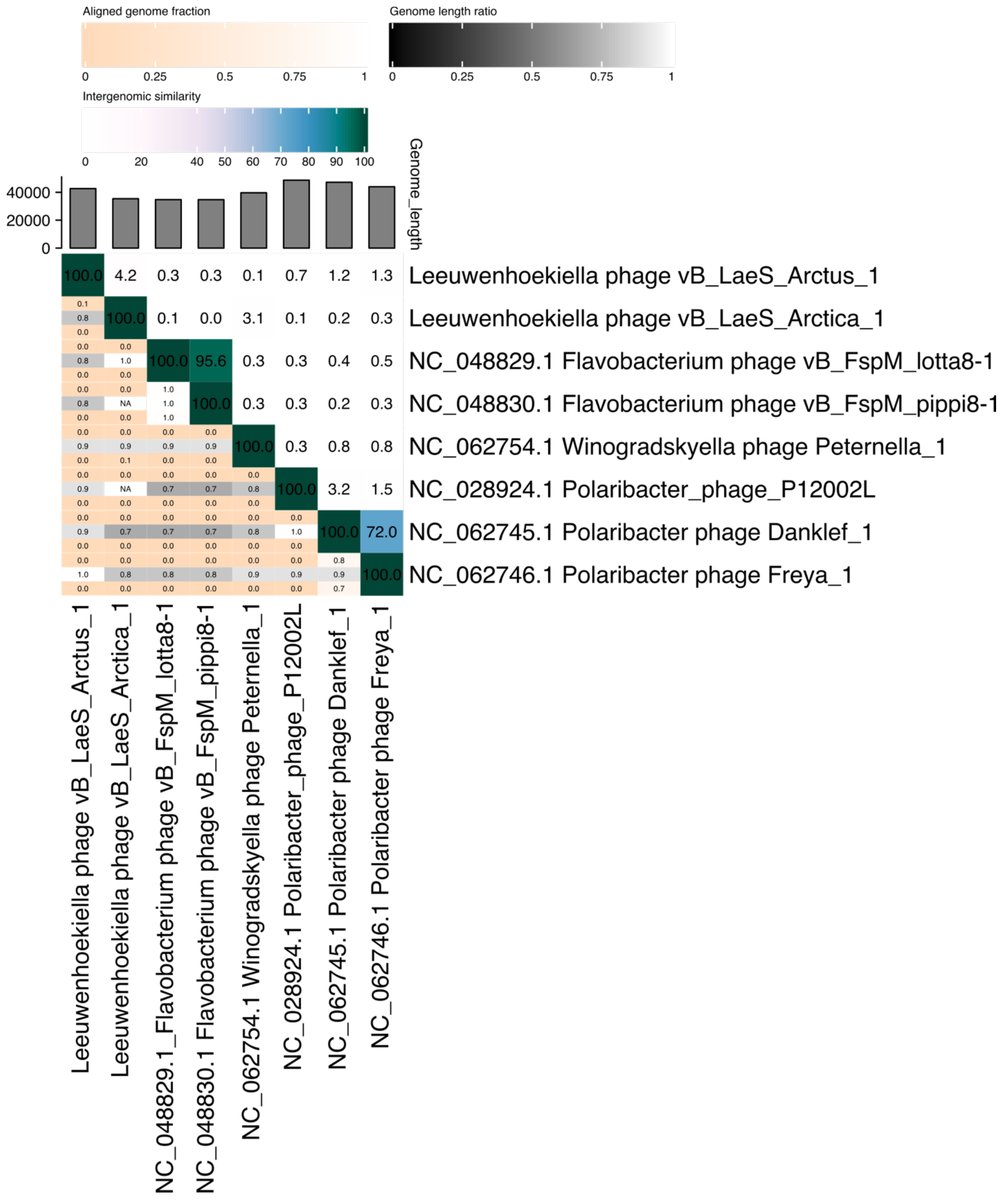

Figure S9: Heatmap derived from VIRIDIC [8] showing low intergenomic similarity between vB_LaeS_Arctus_1 and vB_LaeS_Arctica_1 and related phages as shown in Figure 7d.

Issues with contaminations

We were informed by the sequencing company about possible cross-contaminations in the sequencing plate as the plate's seal broke during shipment, which, after investigation, let us to avoid any inter sample comparisons (abundance, diversity, presence/absence, correlations) for the virome samples 118, 119, 121, 128.

Because we also had evidence for ship-borne water contamination in samples taken from icebreaker Oden's gangway, viruses that were assembled in samples 108, 111, 112, 113 and 128, were excluded from the sample pool unless they formed a species cluster with a vOTU from one of the other "clean" samples. Due to this issue, we also excluded bacterial MAGs from metabolic analysis if according to read breadth they were found at the gangway stations or are typical genera of non-marine, anaerobic bacteria like MAGs related to *Propionivibrio* sp., *Ruminococcus bromii, Sulfurospirillum* sp. All exclusions for MAG analysis have been further specified in Supplement Table S2 by labelling potential contaminants with a "C".

>Arctus_1 Tape measure protein [PF20155.2] (db=pfam)

MAKNQFQDAIEKAGLTLQEIEKRWISIDEKILQASKSASKLGKTDFNSAQPKDLNDRLQK
NATYRKQVNAEMKEQERLNKALAAAQAKFYSTQSGTNRQLQQTRFETNQLNAKYREQ
AILSSKLADEYQKQSTRLNMLRREAKAAAAQYGVNSKEAKNLIRDVNKLDASLKKVDA
AVGQHQRSVGNYGKAWQGVGKLMGAAVGAFGVYSAMQIGREIYAEIKAIDGLNKALK
QVTETTESYNQAKGFLGDLSQETGVQIKELTGAYLSFYAAAKNTNLTLEETQDIFRQTA
KAGATLGLSTEQVEGALRALEQMLSKGKVQAEEIRGQLGERLPGAFQILARSIGVSTAEL
DDMLKKGEVIADEVLPRFARELEKTFSLDKIDKVNTLAAAEGRLSTEWTRFVETLSNED
GAVTGFLTGTLELVTGIVSELRKLNEDLAPKSTSRVFDETIAKYKELGEAGKEEAMNQK
RNSEQAIKDAKNLQVILEERKNKLEEESGWFQNNFGKTKKEYKDLEDSIYNNNAKIAVQ
NGLLQAATEYLGLNTKEVEGNSKAEDENNDKKKKGIIALQGSIGAMEAVISKLEEEQSK
LATNGKEWSEYAGQINKAKDALQKIKTEYEGIEVLFEQEGVKDFDPFDYDLMDESQKR
ALKQVQDNAKAATNLLSDEYEKRLSTLEQFEKRKEDVLRDAARLEFDIRRDAFEKIADT
GQGFFQIEIDRYDQQIDALNENYDAQIEAAEGNEKQQQALRDEKLLKEQELERKKEEAE
KNAFLFSQGLALAQIGIDLARTISAIQVAATAMDALTPFAFGATGTTYRAANIPVAIGTA
AAQTALIAAQTIPALEQGDTTGKHEGQVMINDAKGAKFREIVQRTSGQMEVYSGRNVVI

DKKRGDKVYKAGQAPGGFDYNDLVNASINMSLADQYGRMSQAEAVQTFDFSAMESM
MDRKLSEFTKAVKTNKTVVNVPDSGASFAKAMRLNKIINK

References

1. Chaumeil PA, Mussig AJ, Hugenholtz P, Parks DH. GTDB-Tk v2: memory friendly classification with the genome taxonomy database. Bioinformatics. 2022;38(23):5315-6.
2. Matsen FA, Kodner RB, Armbrust EV. pplacer: linear time maximum-likelihood and Bayesian phylogenetic placement of sequences onto a fixed reference tree. BMC Bioinformatics. 2010;11:538.
3. Huson DH, Scornavacca C. Dendroscope 3: an interactive tool for rooted phylogenetic trees and networks. Syst Biol. 2012;61(6):1061-7.
4. Rambaut A. FigTree. Tree figure drawing tool. http://treebioedacuk/software/figtree/. 2009.
5. Richter M, Rossello-Mora R, Oliver Glockner F, Peplies J. JSpeciesWS: a web server for prokaryotic species circumscription based on pairwise genome comparison. Bioinformatics. 2016;32(6):929-31.
6. Chklovski A, Parks DH, Woodcroft BJ, Tyson GW. CheckM2: a rapid, scalable and accurate tool for assessing microbial genome quality using machine learning. Nat Methods. 2023.
7. Nishimura Y, Yoshida T, Kuronishi M, Uehara H, Ogata H, Goto S. ViPTree: the viral proteomic tree server. Bioinformatics. 2017;33(15):2379-80.
8. Moraru C, Varsani A, Kropinski AM. VIRIDIC-A novel tool to calculate the intergenomic similarities of prokaryote-infecting viruses. Viruses. 2020;12(11).
